# Verapamil Inhibits TRESK (K_2P_18.1) Current in Trigeminal Ganglion Neurons Independently of the Blockade of Ca^2+^ Influx

**DOI:** 10.3390/ijms19071961

**Published:** 2018-07-04

**Authors:** Hyun Park, Eun-Jin Kim, Ji Hyeon Ryu, Dong Kun Lee, Seong-Geun Hong, Jaehee Han, Jongwoo Han, Dawon Kang

**Affiliations:** 1 Department of Neurosurgery, College of Medicine and Institute of Health Sciences, Gyeongsang National University, Jinju 52727, South Korea; 1coo3004@naver.com; 2 Department of Physiology and Institute of Health Sciences, College of Medicine, Gyeongsang National University, Jinju 52727, South Korea; eunjin1981@hanmail.net (E.-J.K.); wlgus9217@naver.com (J.H.R.); dklee@gnu.ac.kr (D.K.L.); hong149@gnu.ac.kr (S.-G.H.); jheehan@gnu.ac.kr (J.H.); 3 Department of Convergence Medical Science, Gyeongsang National University, Jinju 52727, South Korea

**Keywords:** background potassium channel, calcium, trigeminal ganglions, verapamil

## Abstract

Tandem pore domain weak inward rectifier potassium channel (TWIK)-related spinal cord K^+^ (TRESK; K_2P_18.1) channel is the only member of the two-pore domain K^+^ (K_2P_) channel family that is activated by an increase in intracellular Ca^2+^ concentration ([Ca^2+^]_i_) and linked to migraines. This study was performed to identify the effect of verapamil, which is an L-type Ca^2+^ channel blocker and a prophylaxis for migraines, on the TRESK channel in trigeminal ganglion (TG) neurons, as well as in a heterologous system. Single-channel and whole-cell currents were recorded in TG neurons and HEK-293 cells transfected with mTRESK using patch-clamping techniques. In TG neurons, changes in [Ca^2+^]_i_ were measured using the fluo-3-AM Ca^2+^ indicator. Verapamil, nifedipine, and NiCl_2_ inhibited the whole-cell currents in HEK-293 cells overexpressing mTRESK with IC_50_ values of 5.2, 54.3, and >100 μM, respectively. The inhibitory effect of verapamil on TRESK channel was also observed in excised patches. In TG neurons, verapamil (10 μM) inhibited TRESK channel activity by approximately 76%. The TRESK channel activity was not dependent on the presence of extracellular Ca^2+^. In addition, the inhibitory effect of verapamil on the TRESK channel remained despite the absence of extracellular Ca^2+^. These findings show that verapamil inhibits the TRESK current independently of the blockade of Ca^2+^ influx in TG neurons. Verapamil will be able to exert its pharmacological effects by modulating TRESK, as well as Ca^2+^ influx, in TG neurons in vitro. We suggest that verapamil could be used as an inhibitor for identifying TRESK channel in TG neurons.

## 1. Introduction

Tandem pore domain weak inward rectifier potassium channel (TWIK)-related spinal cord K^+^ (TRESK) channel is a member of the two-pore domain K^+^ (K_2P_) channel family that sets the resting membrane potential in excitable and non-excitable cells. Of the K_2P_ channels, TRESK is known as the only member activated by an increase in the intracellular Ca^2+^ concentration ([Ca^2+^]_i_) [1]. Increased [Ca^2+^]_i_ activates calcineurin, a calcium-activated serine–threonine phosphatase, and the calcineurin dephosphorylates TRESK to activate it. The Ca^2+^-induced activation of mouse and human TRESK depends on direct targeting of calcineurin to the nuclear factor of activated T-cell-like consensus motif [2,3].

An increase in the Ca^2+^ influx by ionomycin, an effective Ca^2+^ ionophore, activates the TRESK channel [4]. If the TRESK channel is affected by extracellular Ca^2+^ levels, a blockade of the Ca^2+^ influx through Ca^2+^ channels will inhibit the activity of the TRESK channel. Few studies reported the inhibitory effect of Ca^2+^ channel blockers, such as mibefradil, nimodipine, nitredipine, and nicadipine, on TRESK channels in heterologous system [5,6].

Modulation of K^+^ channels and Ca^2+^ channels expressed in trigeminal ganglion (TG) neurons has been implicated in migraine [7,8,9,10,11]. Opening of K^+^ channels leads to hyperpolarization of the cell membrane and a consequent reduction in cell excitability [9,12,13]. Decrease in excitability of TG neurons may help to control migraines [10]. Blockade of Ca^2+^ channels reduces the release of glutamate and calcitonin gene-related peptide (CGRP), important neurotransmitters in migraines, in TG neurons [7]. It is known that Ca^2+^ channel blockers help to alleviate risk of migraine headache [14,15].

Recent studies have demonstrated that the TRESK channel is linked to migraines [16,17], and that the excitability of TG neurons is modulated by TRESK channel activity [18,19]. However, TRESK channel is blocked by Ca^2+^ channel blockers. Ca^2+^ channel blockers, such as nifedipine and verapamil, are effective migraine prophylactic agents [20,21]. These reports led us to study the effect of Ca^2+^ channel blockers with migraine prophylactic action on TRESK channel expressing in TG neurons. Identifying the effect of Ca^2+^ channel blockers on migraine-related ion channels in TG neurons is needed to understand the relationship between the excitability of TG neurons and migraine. This study was performed to identify the effect of Ca^2+^ channel blockers, particularly nifedipine and verapamil, that are used in the prophylaxis of migraines, on TRESK channels in HEK-293 cells and rat TG neurons.

## 2. Results

### 2.1. Inhibitory Effect of Ca^2+^ Channel Blockers on TRESK Channel Overexpressed in HEK-293 Cells

**Figure 1:**
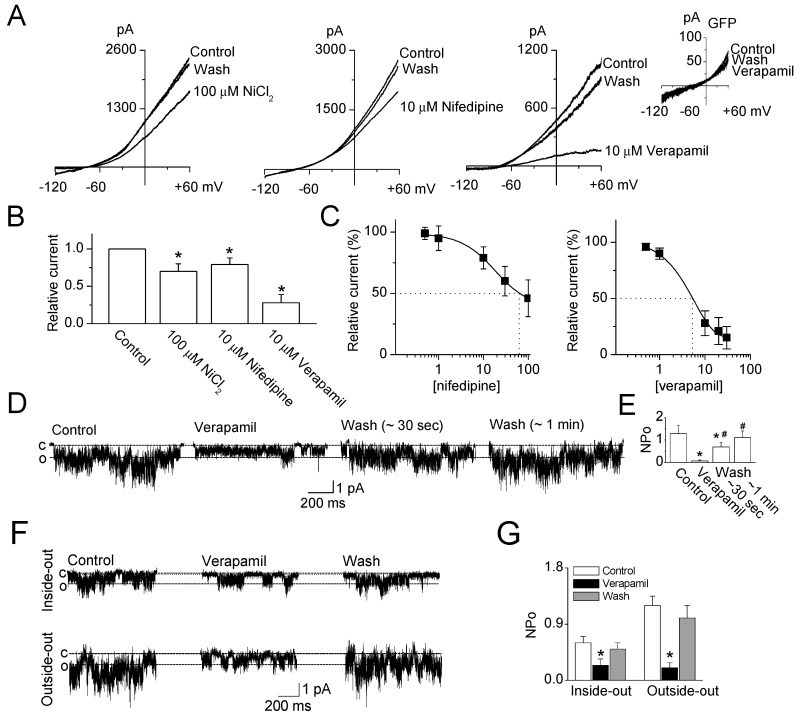
Effect of Ca^2+^ channel blockers on TRESK channels overexpressed in HEK-293 cells (**A**) TRESK whole-cell currents were inhibited by NiCl_2_ (100 μM), nifedipine (10 μM), and verapamil (10 μM). The currents were recorded from HEK-293 cells transfected with mTRESK. GFP represents the currents recorded from only green fluorescent protein (GFP)-transfected cells; (**B**) Summary of the effects of NiCl_2_, nifedipine, and verapamil on the TRESK currents. The current levels at +60 mV were determined and analyzed. Data represent the mean ± SD of three independent transfections (*n* = 10). * *p* < 0.05 compared with the control currents; (**C**) Inhibition curve of nifedipine and verapamil. IC_50_ is the concentration that reduces TRESK currents by 50%. Data represent the mean ± SD of three independent transfections (*n* = 5); (**D**) Verapamil-induced inhibition of TRESK single-channel currents. Single-channel openings were recorded at −60 mV in symmetrical 150 mM K^+^ solution. Verapamil dissolved in DMSO was applied to the cells under cell-attached patches. The data was taken 1 min after each treatment; (**E**) Summary of the effect of verapamil on TRESK channels in the cell-attached modes. Data represent the mean ± SD of three independent transfections (*n* = 6). * *p* < 0.05 compared with the control. ^#^ *p* < 0.05 compared with the verapamil treatment; (**F**) Effect of verapamil on TRESK activity in inside-out and outside-out patches at −60 mV in symmetrical 150 mM K^+^ solution. An outside-out patch was made from a whole-cell patch, which was obtained from cell-attached patch by applying gentle suction. An inside-out patch was directly obtained from a cell-attached patch without process making whole-cell patch; and (**G**) Summary of the effect of verapamil on TRESK channels in the excised patch modes. Data represent the mean ± SD of three independent transfections (*n* = 5). * *p* < 0.05 compared with the control. C and O represent closed and open states, respectively.

The application of Ca^2+^ channel blockers (NiCl_2_, nifedipine, and verapamil) to TRESK channels overexpressed in HEK-293 cells inhibited whole-cell currents, and the response to Ca^2+^ channel blockers was reversible (Figure 1A). NiCl_2_ (100 μM), nifedipine (10 μM), and verapamil (10 μM) inhibited TRESK currents by 30 ± 10%, 21 ± 9%, and 72 ± 11%, respectively (Figure 1B, *n* = 10). NiCl_2_ at 10 μM had no effect on TRESK current. Nifedipine and verapamil were more potent to TRESK than NiCl_2_. Treatment with 0.5, 1, 10, 20, and 30 μM verapamil inhibited the TRESK currents by 4 ± 3%, 10 ± 5%, 72 ± 11%, 79 ± 12%, and 85 ± 10%, respectively. The IC_50_ values of verapamil and nifedipine were 5.2 and 63.8 μM, respectively (Figure 1C, *n* = 5). Nifedipine showed the IC_50_ value with maximal inhibition. As shown in Figure 1D,E, TRESK channel activity (NP_o_, see the Mateials and Methods 4.6.) was significantly inhibited by 10 μM verapamil in cell-attached patches (*p* < 0.05, NP_o_, 1.3 to 0.1, *n* = 6). The verapamil effect was fully lost approximately 1 min after washout. The TRESK channel activity was also inhibited by verapamil under excised patch modes, and the amount of inhibition observed in outside-out patch was higher than that observed in inside-out patch (Figure 1F,G, *n* = 5). The multiple openings (over three levels) were reduced to one or two levels opening by verapamil treatment (Figure 1D,F).

**Figure 2:**
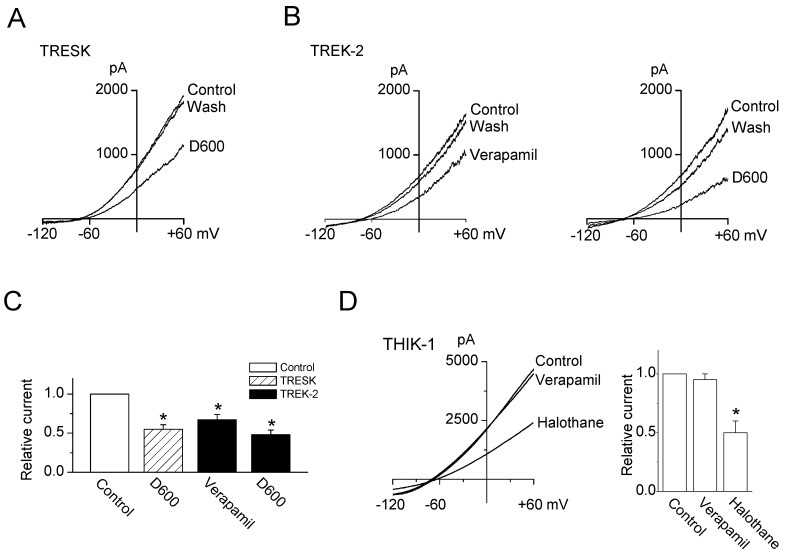
Comparison of verapamil and D600 effects on TRESK and TREK-2 channels (**A**) Effect of D600 on TRESK; (**B**) Effect of verapamil and D600 on TREK-2; (**C**) Summary of the effects of verapamil and D600 on the TRESK and TREK-2 currents. The current levels at +60 mV were determined and analyzed. Data represent the mean ± SD of three repeated experiments. * *p* < 0.05 compared with the control; and (**D**) Effect of verapamil on THIK-1 current. The THIK-1 current was validated by treatment with halothane. Data represent the mean ± SD of three repeated experiments. * *p* < 0.05 compared with the control.

D600 (methoxyverapamil, 20 μM), an analogue of verapamil, also inhibited TRESK currents by 45 ± 6% (Figure 2A,C). In addition, both verapamil and D600 inhibited TREK-2 currents expressed in HEK-293 cells by 33 ± 7% and 52 ± 6%, respectively. The inhibitory effects of verapamil and D600 on TREK-2 were lower and higher than those on TRESK, respectively (Figure 2B,C). Unlike TRESK and TREK-2, verapamil had no effect on THIK-1 channel, which is expressed in TG neurons. The THIK-1 currents were strongly inhibited by halothane (1 mM), a well-known inhibitor of THIK-1 (Figure 2D).

### 2.2. Verapamil Inhibits TRESK-Like Currents in TG Neurons Independently of the Blockade of Ca^2+^ Influx

**Figure 3:**
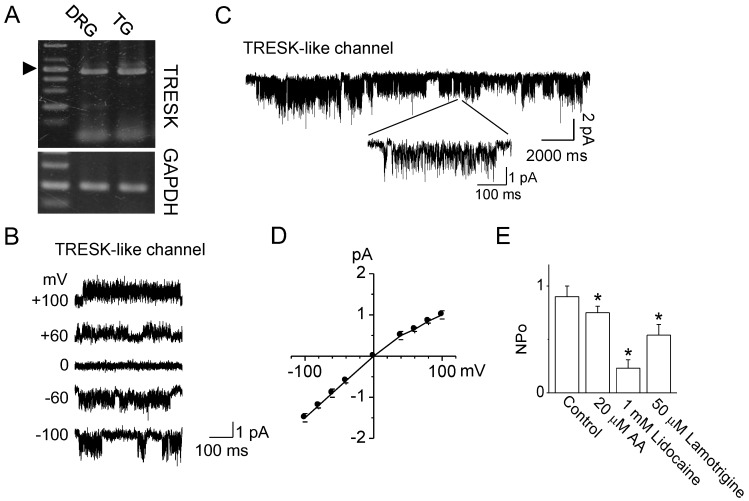
Functional expression of TRESK channels in rat TG neurons. (**A**) TRESK mRNA expression in TG neurons. DRG neurons were used as a positive control for TRESK expression. GAPDH was used as a loading control. An arrow head indicates 500-bp marker; (**B**) Single-channel recording of TRESK-like channel in TG neurons. Various pipette potentials (+100 to −100 mV) were applied to TG neurons in symmetrical 150 mM K^+^ solution; (**C**) Single-channel openings were recorded at 0 mV (pipette potential) in 5 mM K^+^ solution; (**D**) Current–voltage relationship was drawn using amplitude recorded in various voltages; and (**E**) Effect of TRESK blockers on TRESK-like single-channel currents expressed in TG neurons. Data represent the mean ± SD of three repeated experiments. * *p* < 0.05 compared with the control.

The expression of TRESK in rat TG neurons was examined to identify the effect of verapamil on TRESK expressed in primary cells. As shown in Figure 3A, TG neurons express TRESK mRNA (475 bp). Dorsal root ganglion (DRG) neurons were used as a positive control for TRESK detection. Single-channel data were recorded in 150 mM KCl in the pipette and bath solutions under cell-attached mode (Figure 3B, *n* = 5). The channel was active at all membrane potentials tested (−100 mV to +100 mV). A single-channel opening obtained with a pipette potential of 0 mV in the 5 mM KCl bath solution and 150 mM KCl pipette solution is shown on an expanded scale (Figure 3C). The current–voltage relationship, which was plotted using the mean amplitude values, showed that TRESK-like channels produce a slightly inward rectification (Figure 3D, *n* = 5). The conductance was 13.3 ± 0.6 pS and 16 ± 1.0 pS at +60 mV and −60 mV, respectively. Arachidonic acid (AA), lidocaine, and lamotrigine inhibited the activity of TRESK-like channel by 25 ± 6%, 77 ± 8%, and 46± 10%, respectively, in the cell-attached patches (Figure 3E, *n* = 5).

**Figure 4:**
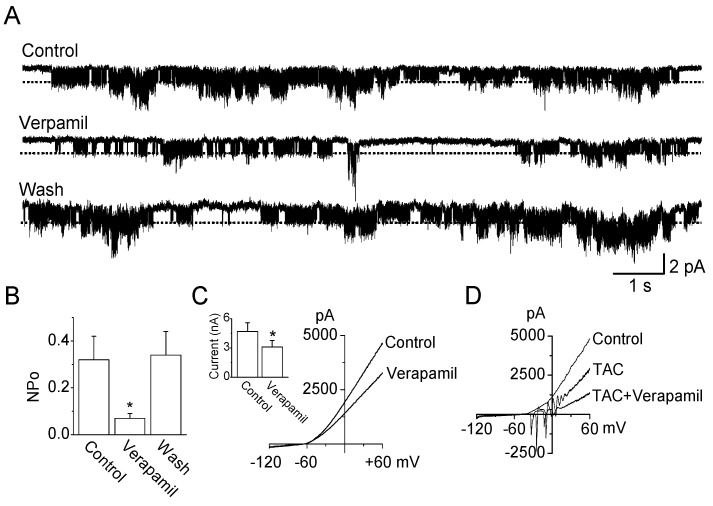
Effect of verapamil on the TRESK-like channel expressed in TG neurons. (**A**) The TRESK-like single-channel current was inhibited by the application of verapamil (10 µM). Single-channel openings were recorded at 0 mV (pipette potential) in 5 mM K^+^ solution; (**B**) Summary of the effects of verapamil on single-channel currents recorded from TG neurons. Data represent the mean ± SD of three repeated experiments. * *p* < 0.05 compared with the control; (**C**) Whole-cell currents were inhibited by verapamil. The current levels at +60 mV were determined and analyzed. Data represent the mean ± SD of three repeated experiments. * *p* < 0.05 compared with the control; and (**D**) Effect of verapamil on whole-cell currents recorded from TG neurons in the presence of voltage-dependent K^+^ channel blockers. TAC represents combination of TEA, 4-AP, and CsCl.

In cultured TG neurons, verapamil (10 μM) inhibited the activity of TRESK-like channel by 76 ± 10% in cell-attached patches (Figure 4A,B, *n* = 6). In outside-out patches, TRESK-like channel activity was also inhibited by 70 ± 8%. The inhibitory effect of verapamil on TRESK expressed in TG neurons was similar to that observed in HEK-293 cells. However, in whole-cell current, verapamil effect was small. Verapamil inhibited the whole-cell currents recorded from TG neurons by approximately 30% (Figure 4C). The percentage of inhibition by verapamil was constant in the absence and presence of K^+^ channel blockers (TAC; combination of tetraethyl ammonium (TEA), 4-AP, and CsCl), which do not affect the TRESK channels (Figure 4D; TAC to TAC + Verapamil). Figure 4D showed whole-cell currents with spontaneous depolarizations in response to ramp voltage pulses in agreement with a previous study performed in TG neurons [22]. The whole-cell currents were reduced by TAC, and TAC-inhibited currents were reduced by verapamil treatment.

Resting membrane potentials (RMP) were measured to identify whether verapamil-induced blockade of TRESK channel depolarizes the RMP in TG neurons. Verapamil treatment depolarized the RMP of both HEK293 cells overexpressed with TRESK and TG neurons (HEK293 cells: −77.6 ± 2.5 to −68.4 ± 3.3 mV, *p* = 0.012; TG neurons: −63.8 ± 4.1 to −56.5 ± 2.4 mV, *p* = 0.006).

**Figure 5:**
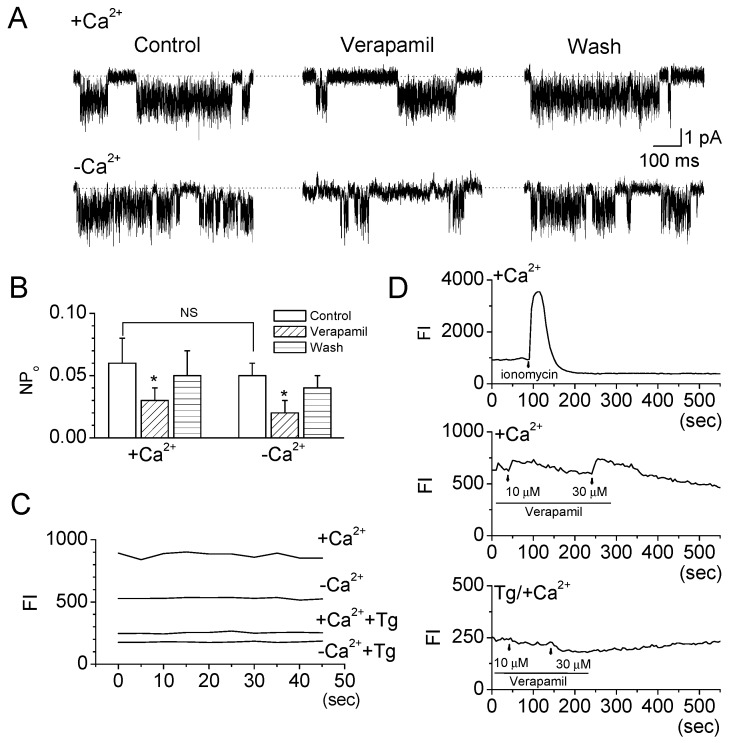
Effect of extracellular Ca^2+^ on the inhibition of TRESK channel activity by verapamil. (**A**) Effect of extracellular Ca^2+^ on verapamil effect. The channel activity was recorded in 5 mM KCl solution at 0 mV with and without extracellular Ca^2+^. The extracellular Ca^2+^-free solution contains 0 mM Ca^2+^ and 5 mM EGTA; (**B**) Summary of the effects of verapamil on single-channel currents in the presence and absence of extracellular Ca^2+^. Data represent the mean ± SD of three repeated experiments. * *p* < 0.05 compared with the corresponding control; (**C**) Basal Ca^2+^ levels of TG neurons in the presence and absence of extracellular Ca^2+^ and in the depletion of Ca^2+^ from endoplasmic reticulum with thapsigargin (Tg). Tg (10 μM) was applied to TG neurons for 10 min before the recording of the basal Ca^2+^ level; and (**D**) Effect of verapamil on the basal Ca^2+^ level in TG neurons in the presence of extracellular Ca^2+^ and Tg. FI: fluorescence intensity (arbitrary units) of the cells. +: presence of extracellular Ca^2+^, –: absence of extracellular Ca^2+^.

To identify the effect of extracellular Ca^2+^ level on the TRESK channel activity in TG neurons, the TRESK current was recorded in the presence and absence of extracellular Ca^2+^ under cell-attached patches. Its activity tended to decrease in the absence of extracellular Ca^2+^ (Figure 5A, *n* = 5), but this was not significant (Figure 5B). Verapamil inhibited the activity of the TRESK channel even in the absence of extracellular Ca^2+^ (0.05 ± 0.01 to 0.02 ± 0.01, Figure 5A,B). The percentage of inhibition was similar between the presence and absence of extracellular Ca^2+^ (Figure 5B, *n* = 5). The basal intracellular Ca^2+^ level in the TG neurons was lower in the absence of extracellular Ca^2+^ than in the presence of extracellular Ca^2+^ (*n* = 5). Treatment with thapsigargin, which is used to deplete the Ca^2+^ stores in the endoplasmic reticulum and reduce intracellular Ca^2+^ levels, showed low basal intracellular Ca^2+^ levels (Figure 5C). As shown in Figure 5D, verapamil treatment slightly increased the basal intracellular Ca^2+^ level in the TG neurons. Verapamil (10 μM) showed very small effect on the intracellular Ca^2+^ level (*n* = 5). However, in TG neurons pretreated with thapsigargin, the intracellular Ca^2+^ level was decreased by verapamil. Treatment with ionomycin (5 μM) highly increased the intracellular Ca^2+^ level (Figure 5D), but it did not affect TRESK channel activity significantly in TG neurons (*p* = 0.074).

## 3. Discussion

This study shows an advance in the understanding the mechanism of action of verapamil on TRESK channel. We first assumed that Ca^2+^ channel blockers could inhibit TRESK activity, because it is known that the TRESK channel is activated by an increase in [Ca^2+^]_i_ [1,2]. As expected, TRESK currents were inhibited by treatments with verapamil and compound D600, an analogue of verapamil. However, the regulatory mechanism of verapamil on TRESK channel was different from what we hypothesized.

### 3.1. Direct Inhibition of TRESK Currents by Verapamil

The TRESK currents seem to be directly blocked by verapamil rather than through blockade of Ca^2+^ influx. Verapamil showed an inhibitory effect on TRESK currents despite the absence of extracellular Ca^2+^. In addition, TRESK was inhibited by verapamil under inside-out and outside-out patch modes. These results indicate that verapamil inhibits TRESK current independently of the blockade of Ca^2+^ influx. Similar to TRESK channels, Kv channels expressed in smooth muscle cells isolated from a rabbit coronary artery are inhibited by verapamil independently of any Ca^2+^ channel inhibition [23]. Verapamil is known to block many types of K^+^ channels, such as ATP-sensitive K^+^ channel [24], inactivating delayed K^+^ channel [25], Kv1.1, Kv1.5, Iks, hERG [26], and Kv1.3 [27]. However, the mechanisms are not fully addressed. Verapamil diffuses out into cells in the neutral form and acts at a site within the pore domain via hydrophobic pathways [25,28], and directly binds to sites within the pore domain or at the inner S6 helix of hERG channels [28,29]. Verapamil could also bind to sites within the pore domain and at cytoplasmic loop and N- and C-terminus of TRESK, like verapamil does in hERG channel. Inhibitory effect of verapamil on TRESK channel was higher than that of nifedipine. Nifedipine does not block hERG current [28]. The binding site and regulatory mechanism of verapamil and nifedipine will be different in the TRESK channel. Verapamil and nifedipine bind to different sites of L-type Ca^2+^ channel. Verapamil binds to the α1 site in the near C-terminal chain and the S6 helix, whereas nifedipine binds Ca^2+^ channel pore (dihydropyridine binding site) [30].

It has been reported that TRESK activity is markedly affected by the extracellular [Ca^2+^] in a heterologous system [31]. However, in TG neurons, removal of extracellular Ca^2+^ did not significantly affect TRESK channel activity. Treatment with high concentration of Ca^2+^ (5 mM CaCl_2_) or ionomycin to bath solution also failed to increase significantly TRESK activity in TG neurons. Acetylcholine, which induces an increase in [Ca^2+^]_i_, increases the TRESK activity in rat DRG neurons, but the effect was much less than that in a heterologous system [32]. TRESK channels expressed in TG neurons seem to be insensitive to changes in [Ca^2+^]_i_. These differences between DRG and TG could be from changes in gene expression between them. Comprehensive RAN-Seq expression analysis of DRG and TG shows different expression patterns in the ion channels and G protein-coupled receptors (GPCRs) [33]. TG is functionally and anatomically similar to DRG, but TG lacks cell bodies of large-diameter proprioceptors compared to DRG. In addition, the difference between heterologous systems and primary cells could result from different Ca^2+^ control systems present in each cell.

### 3.2. Functional Expression of TRESK in TG Neurons

Compared to DRG neurons, relatively few studies have reported the expression of TRESK in TG neurons. Most of studies demonstrated the expression of TRESK in TG neurons without single-channel recording [18,19,34]. Only Callejo et al. (2013) reported the single-channel kinetics of TRESK in TG neurons [35]. Our study demonstrates the functional expression of TRESK channel in TG neurons by recording single-channel currents and analyzing inhibition by 20 μM AA, 1 mM lidocaine, and 50 μM lamotrigine, which were confirmed as TRESK blockers in a heterologous system [32,34,36].

TG neurons express many subtypes of K_2P_ channels, including THIK-1, TREK-2, and TRESK [18,19,22,34,35,37,38], which are expressed in DRG neurons. They act as main background K^+^ channel in DRG and TG neurons. Rat DRG and mouse superior cervical ganglion strongly express TRESK and TREK-2 [39,40], but little is known about their expression levels in TG neurons. The whole-cell currents in TG neurons were inhibited by approximately 30% by verapamil in a bath solution containing 1 mM TEA, 1 mM CsCl, and 1 mM 4-AP, which do not affect K_2P_ channels. This inhibition is similar to TRESK contribution percentage to the background K^+^ currents in DRG neurons estimated by Dobler et al. (2007) [41]. If TRESK and TREK-2 are main background K^+^ channels in TG neurons, the whole-cell currents should be more inhibited by verapamil than 30%, because verapamil inhibits both TRESK and TREK-2 channels. The expression levels of TRESK and TREK-2 were not compared in this study. At 24 °C and 37 °C, TRESK contributes 96% and 16% to the background K^+^ conductance in DRG neurons, respectively. At 37 °C, TRESK and TREK-2 together contribute approximately 95% of the background K^+^ conductance of DRG neurons [40]. This result indicates that expression levels of TRESK and TREK-2 in TG neurons might be lower than those in DRG neurons. These differences could be from different species (rat and mouse), cell types (TG and DRG), and age of animal (postnatal day 1/2 and 14).

### 3.3. Physiological Role of TRESK Inhibition by Verapamil

Verapamil inhibited TRESK currents at low concentration of IC_50_ 5.2 μM. However, the concentration is higher than that of therapeutic concentration of verapamil, as reported by U.S. Food and Drug Administration. Chronic oral administration of the 120 mg every 6 hours ranges from 125 to 400 ng/mL in plasma verapamil levels. The therapeutic concentration of verapamil is approximately 1 μM. TRESK currents were slightly inhibited at 1 μM of verapamil. Verapamil would not be expected to inhibit TRESK much in vivo. However, in vitro, verapamil could be used as a compound to discriminate TRESK channel from other ion channels in primary cells, such as DRG and TG neurons.

Until now, few compounds are known to inhibit TRESK channel activity. Hydroxyl-α-sanshool, a pungent agent from Szechuan peppers, inhibits TRESK currents with an IC_50_ of ~52 μM [42]. Lamotrigine, a Na^+^ channel blocker, and IBA, an alkylamide derivative of hydroxyl-α-sanshool, inhibit TRESK currents with an IC_50_ of 47 μM and ~500 μM, respectively [32,43]. Loratadine, an antihistamine, also inhibits TRESK activity with an IC_50_ of 490 nM [6]. Compared to other compounds, the IC_50_ of verapamil is low. Verapamil could be included in the compounds that inhibit TRESK activity. In addition to verapamil, mibefradil, a T-type and L-type Ca^2+^ channel blocker, also shows a low IC_50_ of 2.2 μM [5,6]. These results indicate that TRESK might be sensitive to Ca^2+^ channel blockers. The precise regulatory mechanism needs to be addressed.

Earlier studies have suggested that activation of TRESK channel might be helpful for the treatment of migraine [16,17,18,19]. However, this channel is inhibited by Ca^2+^ channel blockers, which alleviate migraines. Blockade of TRESK and L-type Ca^2+^ channels could cause different effects on the excitability of TG neurons. TRESK blockade increases the cell excitability, but the blockade of the l-type Ca^2+^ channel reduces the cell excitability. These conflicting consequences could result in no gain or loss in the excitability of TG neurons in response to verapamil, that is, there is no overall change. However, the change could depend on the expression level of targets for verapamil. In addition, at rest, verapamil is likely to block TRESK channel rather than Ca^2+^ channel, because TRESK channels are opened at resting state, and Ca^2+^ channels are closed. Identifying the effect of migraine prophylactic agents on ion channels expressed in TG neurons is very important to understand pathophysiology of migraine in terms of TG neurons’ excitability.

## 4. Materials and Methods

### 4.1. Ethical Approval

All experiments were performed with the approval of the Ethics Committee of Gyeongsang National University (GLA-140510-R0086, 10.05.2014).

### 4.2. Chemicals

All of the chemicals used in this study were purchased from Sigma Chemical Company (St. Louis, MO, USA) unless otherwise specified. Stock solutions of nifedipine (50 mM), D600 (100 mM), and lamotrigine (100 mM) were prepared in dimethyl sulfoxide (DMSO), and then diluted in experimental solution to a working concentration. Lidocaine (50 mM) and verapamil (50 mM) were prepared in ethanol. NiCl_2_ (100 mM) was dissolved in distilled water. When DMSO or ethanol was used as a solvent, a solution containing an equivalent concentration was used as a control. Saturated halothane solution (~18 mM) and arachidonic acid (AA, 20 mM) were dissolved in recording solution, diluted to desired concentrations, and ultrasonicated for 5 min in ice just before use.

### 4.3. Animal Care

Sprague Dawley rats were purchased from Koatech Co. (Animal Breeding Center, Pyeongtaek, Korea). Animal experiments were performed in accordance with the guidelines of the Gyeongsang National University Animal Care and Use Committee (GLA-140510-R0086). The rats were housed under a 12 h light/dark cycle in a pathogen-free area, with food and water freely available.

### 4.4. Culture of TG Neurons

The culture of TG neurons was performed as described previously [22]. In brief, ganglia were dissected from the brain of 2-week-old rats (*n* = 10) and collected in culture medium. Ganglia were incubated for 1.5 h at 37 °C in the F-12 medium containing 0.125% collagenase (Type II). The ganglia were then incubated in F-12 medium containing 0.25% trypsin for 30 min at 37 °C. The digested tissue pieces were then placed in DMEM medium containing 10% fetal bovine serum (FBS) and 0.1% streptomycin/penicillin and gently triturated with a polished glass pipette tip. The suspended cells were plated on poly-l-lysine-coated glass coverslips in a culture dish. Cells were incubated at 37 °C in a 95% air–5% CO_2_ gas mixture, and used 1 day after plating.

### 4.5. Transfection

HEK-293 cells were seeded at a density of 2 × 10^5^ cells per 35 mm dish 24 h prior to transfection in Dulbecco’s modified Eagle’s medium (DMEM) containing 10% FBS. HEK-293 cells were co-transfected with DNA fragments encoding mouse TRESK (NM_207261) or rat TREK-2 (NM_023096) and green fluorescent protein (GFP) in pcDNA3.1 using LipofectAMINE2000 and OPTI-MEM I reduced serum medium (Life Technologies, Grand Island, NY, USA). Green fluorescence from cells expressing GFP was detected with the aid of a Nikon microscope equipped with a mercury lamp light source. Cells were used 2–3 days after transfection.

### 4.6. Electrophysiological Studies

Electrophysiological recording was performed using a patch clamp amplifier (Axopatch 200, Axon Instruments, Union City, CA, USA). Single-channel currents were filtered at 2 kHz using an 8-pole Bessel filter (−3 dB; Frequency Devices, Haverhill, MA) and transferred to a computer (Samsung, Suwon, Korea) using the Digidata 1320 interface (Axon Instruments, Union City, CA, USA) at a sampling rate of 20 kHz. Threshold detection of channel openings was set at 50%. Single-channel currents were analyzed with the pCLAMP program (version 10, Axon). The filter dead time was 100 μs (0.3/cutoff frequency) for single-channel analysis, therefore, events lasting less than 50 μs were not detected. Channel activity (NP_o_, where N is the number of channels in the patch and P_o_ is the probability of a channel being open) was determined from ~1–2 min of current recording. The single-channel current tracings shown in the figures were filtered at 2 kHz. In experiments using cell-attached and excised patches, the pipette and bath solutions contained (mM): 150 KCl, 1 MgCl_2_, 5 EGTA, and 10 HEPES (pH 7.3). The pH was adjusted to desired values with HCl or KOH. For whole-cell currents, bath solution contained (mM): 135 NaCl, 5 KCl, 1 CaCl_2_, 1 MgCl_2_, 5 glucose, and 10 HEPES, and pipette solution contained (mM): 150 KCl, 1 MgCl_2_, 5 EGTA, and 10 HEPES (pH 7.3). All solutions were prepared with Milli-Q water (18.2 MΩ-cm at 25 °C). Whole-cell current was recorded in response to a voltage ramp (−120 to +60 mV; 865 ms duration) from a holding potential of −80 mV. The currents measured at +60 mV were analyzed. All experiments were performed at ~25 °C.

### 4.7. Reverse Transcriptase-Polymerase Chain Reaction (RT-PCR)

First-strand cDNAs were synthesized from total RNA isolated from DRG and TG neurons using oligo dT (SuperScript^TM^ First-Strand Synthesis System, Invitrogen, Carlsbad, CA, USA) according to the manufacturer’s instructions. Specific primers for rat TRESK (GenBank accession number: AY567970; sense, 5’-CCAGAAGCAGAGGAGAACCC-3’ and antisense, 5’-CTGCACCAGCATCAATGACA-3’) were used for PCR with *Taq* polymerase (Takara, Otsu, Japan). The PCR conditions for RT-PCR were as follows: initial denaturation at 94 °C for 5 min; 30 cycles at 94 °C for 30 s, 55 °C for 30 s, and 72 °C for 30 s; and a final extension step at 72 °C for 10 min. The products were electrophoresed on a 1.2% (*w*/*v*) agarose gel to check product size (475 bp). The DNA fragments obtained by RT-PCR were directly sequenced with the ABI PRISM^®^ 3100-Avant Genetic Analyzer (Applied Biosystems, Foster City, CA, USA).

### 4.8. Measurement of [Ca^2+^]_i_

Changes in [Ca^2+^]_i_ were measured using the Ca^2+^-sensitive fluorescent indicator fluo-3-AM (Molecular Probes, Eugene, OR, USA) and a confocal laser scanning microscope (IX70 Fluoview; Olympus, Tokyo, Japan). TG neurons were incubated with 5 μM Fluo 3-AM in a glass-bottom dish (SPL, Pocheon, South Korea) for 45 min at 37 °C and washed three times with serum-free DMEM without phenol red. [Ca^2+^]_i_ was measured as described previously [44] with slight modification. In each cell studied, fluorescence intensity (FI, arbitrary units) was measured and [Ca^2+^]_i_ was represented as FI.

### 4.9. Statistical Analysis

Differences among groups were analyzed using one-way ANOVA test with post hoc comparisons using Tukey’s test (SPSS18 software, SPSS, Chicago, IL, USA). Data are represented as mean ± SD. A *p* < 0.05 was considered as the criterion for significance.

## 5. Conclusions

In conclusion, verapamil reversibly inhibits TRESK currents in a dose-dependent manner with a low IC_50_ (5.2 μM), independently of the blockade of Ca^2+^ influx. Verapamil will be able to exert its pharmacological effects by modulating TRESK as well as Ca^2+^ influx in TG neurons.

## Abbreviations

DRG	Dorsal root ganglion
TG	Trigeminal ganglion neuron
TRESK	TWIK-related spinal cord K^+^ channel
